# The association between long-term night shift work and metabolic syndrome: a cross-sectional study of male railway workers in southwest China

**DOI:** 10.1186/s12872-022-02705-7

**Published:** 2022-06-11

**Authors:** Chaohui Dong, Honglian Zeng, Bo Yang, Yi Zhang, Zhitao Li

**Affiliations:** 1 Department of Health Management Center, Clinical Medical College and Affiliated Hospital of Chengdu University, Chengdu University, Sichuan Province, Jinniu District, Chengdu City, 610081 China; 2 China Railway Chengdu Group Co., Ltd., Jinniu District, Chengdu City, 610081 Sichuan Province China

**Keywords:** Metabolic syndrome, Night shift work, Occupational health

## Abstract

**Objectives:**

Metabolic syndrome (MetS) increases the risk of new diabetes and cardiovascular disease. Night shift work (NSW) may influence metabolic disturbance and lead to MetS. This study aims to investigate the association between long-term NSW (≥ 10 years) and MetS combined with its components in male railway workers in southwest China.

**Methods:**

11,023 male railway workers with long-term NSW of more than 10 years in the Physical Examination Center of the Affiliated Hospital of Chengdu University were enrolled. The basic data were collected by investigators and blood test results were collected. The primary outcome was the prevalence of metabolic syndrome. The results were analyzed using statistical software SPSS 22.0.

**Results:**

In total, 11,023 people over the age of 40 with more than 10 years of working experience were enrolled, and 4759 (43.2%) participants had a diagnosis of MetS. The basic data indicated that night shift workers tended to be younger, shorter working years, but with higher body mass index and longer hip circumference (*p* < 0.05). The adjusted analysis revealed that there was no significant association between NSW and metabolic syndrome (OR 1.03, 95% CI 0.94–1.12, *p* = 0.543). NSW was associated with SBP ≥ 130 mmHg (OR 1.11, 95% CI 1.02–1.21, *p* < 0.001) and waist circumference ≥ 90 cm (OR 1.11, 95% CI 1.02–1.21, *p* < 0.001).

**Conclusions:**

Long-term night shift workers had a higher prevalence of MetS. However, long-term NSW is not associated with a significantly increased risk of metabolic syndrome in male railway workers in southwest China. Long-term NSW is associated with elevated SBP, and waist circumference increase.

**Supplementary Information:**

The online version contains supplementary material available at 10.1186/s12872-022-02705-7.

## Introduction

Metabolic syndrome (MetS) is a disease syndrome characterized by abdominal obesity, hypertension, hyperglycemia, high triglyceride (TG), and low high-density lipoprotein (HDL), which increased the risk of new diabetes and cardiovascular disease [1, 2]. With the development of the social economy and the improvement of per capita living standards, the incidence of metabolic syndrome is on the rise [3, 4]. Precious studies indicated that people with MetS were at increased risk of cardiovascular events [5], however, there may be gender differences [6, 7]. MetS evaluated BP, plasma glucose, apo B-containing lipoprotein, and inflammatory cytokines, which leads to atherosclerotic plaque development and rupture [8, 9].

Rotating NSW refers to work day and night because of the nature of the jobs, which can lead to sleep rhythm inversion and disturbance [10, 11]. The prevalence of NSW ranged from 20.88%to 45.5% according to different types of work and were higher in women [12–14]. Previous studies showed that occupations such as nursing may require long night shifts, which may associate with a statistically significant but small absolute increase in CHD risk [15, 16]. Other studies concentrated on the association between NSW and MetS, however, the results were inconsistent [6, 7, 12, 14]. Some studies have suggested that NSW and sleep quality may be associated with an increased risk of MetS [12, 14], while others have suggested the opposite, especially in male patients [6, 7]. NSW also influenced the hepatorenal function of night shift workers, studies on Japanese workers showed that shift work and awaking in the night increased the risk of chronic kidney diseases [17, 18], while a 4-year cohort study of 15,871 workers indicated that shift work is associated with hyperuricemia [19]. The previous study has shown that liver function is also affected by NSW [20]. However, previous systematic reviewed retrieved original reviews and found some methodological problems presented in these studies, such as waist circumference being replaced by body mass index [21]. On the other hand, study indicated that the short time of NSW do not increase the risk of CVD [15].

Since most of the previous studies focused on female nurses, there was no accurate description of the length of NSW. We therefor do the study aims to investigate the association between long-term NSW (more than 10 years) and MetS combine with its components in male railway workers in southwest China.

## Methods

### Study population

A cross-sectional survey was conducted in the Physical Examination Center of the Affiliated Hospital of Chengdu University from January 2020 to December 2020. Railway workers from Sichuan, Chongqing, and Guizhou provinces were enrolled in our study.

### Patient and public involvement

All included participants voluntarily participated in this study and signed the informed consent form. This study was approved by the Affiliated Hospital of Chengdu University.

### Inclusion and exclusion criteria

Inclusion criteria: People over 40 years of age and 10 working years were included in this study. Exclusion criteria are as follows: severe hepatic and renal insufficiency; malignant tumor; incomplete basic information or blood test data.

### Data collection

Basic data are collected by the health train, which passes along the railway and conducts health check-ups for employees. The investigators used questionnaires designed by professionals to collect the data, including basic demographic information (age, sex), health behaviors (alcohol consumption, smoking), and history of chronic diseases (hypertension and diabetes, cardiovascular diseases). The body mass index (BMI) was calculated as weight in kilograms (measured to the nearest 0.1 kg by a uniform scale)divided by the square of height in meters (measured to the nearest 0.1 cm by uniform height ruler). Waist circumference was measured to the nearest 0.1 cm at the end of normal expiration on bare skin, midway between the lower rib margin and iliac crest by a uniform flexible rule. Blood pressure readings were taken from the participants’ resting blood pressure in the morning. Blood samples were obtained in the morning, including blood lipid, creatinine (Cr), uric acid (UA), liver function, fasting blood glucose (FBG). The biochemical parameters were measured at the laboratory of the Affiliated Hospital of Chengdu University. All control values were consistent with the standards recommended by the medical laboratory of the China Center for Disease Control and Prevention. All laboratory technicians were trained in formal laboratory biosafety and biosecurity procedures.

### Definitions

The definition of MetS has been updated over time [2], and we used the following definitions according to the American Heart Association [1] (at least 3 of the following 5 risk factors are present): abdominal obesity: waist circumference ≥ 90 cm for men and ≥ 80 cm for women; hypertension: systolic blood pressure (SBP) ≥ 130 mm Hg or diastolic blood pressure (DBP) ≥ 85 mm Hg or current use of antihypertensive medication; hyperglycemia: fasting plasma glucose (FBG) ≥ 100 mg/dl (5.6 mmol/l) or current use of antidiabetic medication; low HDL: HDL < 40 mg/dl (1.04 mmol/l) or current use medication treatment; and hypertriglyceridemia: triglyceride ≥ 150 mg/dl (1.7 mmol/l) or current use of medication treatment for elevated triglyceride. Overweight was defined as a BMI ≥ 24.0 kg/m^2^, according to the cut-off points for Chinese adults. Smoking status was categorized according to 1 year of smoking at least one cigarette per day. Alcohol consumption was considered in terms of whether the participant consumed alcohol at least 12 times in 1 year. Based on previous literature [10, 11], NSW was defined as working during the evening and overnight hours (6 P.M.–8 A.M). The working rhythms were provided by the Chengdu Railway Bureau, the working rhythms varies from different types of job.

### Statistical analysis

All data were analyzed using SPSS statistical software (version 22.0). The means and standard deviations of the continuous variables were provided. Student’s t-test was used to test the differences in the means of the continuous variables, and the chi-square test was used to test the differences in the categorical variables. We did stratified analysis by age and working years. We applied univariate logistic regression models to assess the association of NSW and covariates with MetS, and their odds ratios (OR) and 95% confidence intervals (CI) were estimated. We employed multivariable logistic regression models (model 1) to analyze the association between the independent variable (NSW) and the dependent variable (MetS).Model 2 was adjusted for age, model 3 was adjusted for age and working years, model 4 was adjusted for age, working years, smoking status, alcohol consumption, and previous CAD. We also analyzed the association between NSW and the components of MetS. Bonferroni adjustment was used as post hoc comparisons to adjust for type I error, *p* value below 0.05 (*p* < 0.05) was considered as statistically significant.

## Results

### Baseline characteristics of the study population

**Fig. 1:**
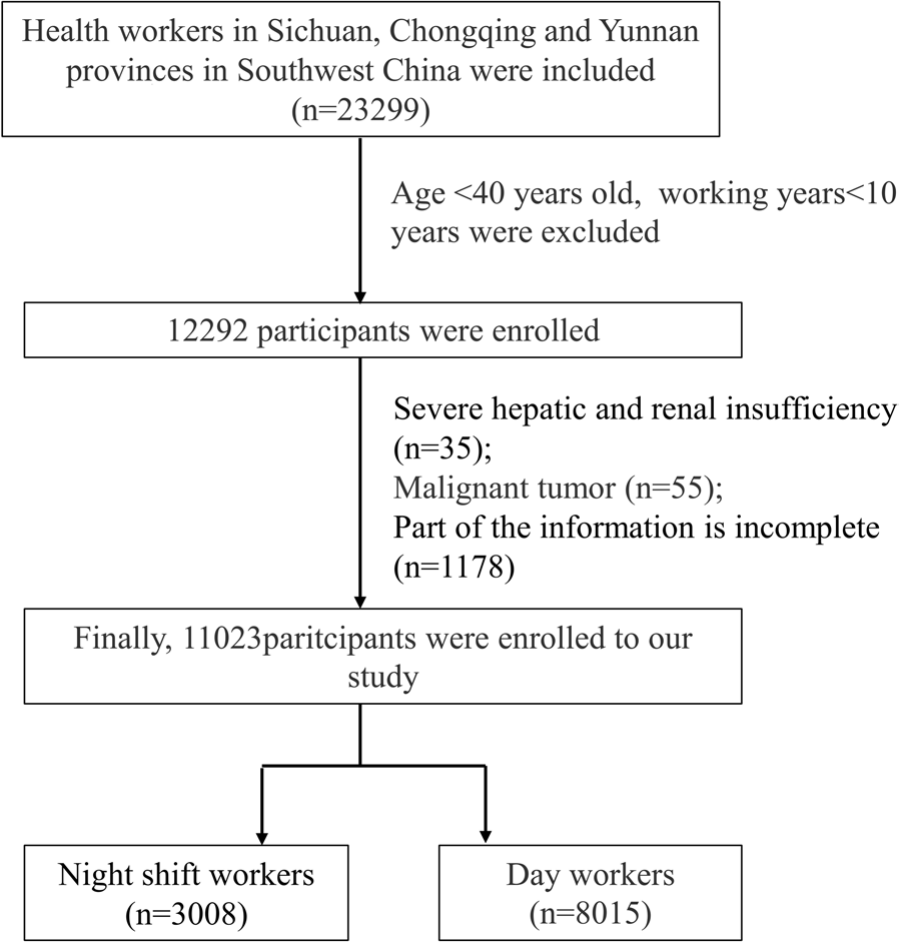
Flow chart of sample inclusion and exclusion

In total, 11,023 participants including 3008 night shift workers were enrolled in the study (Fig. 1). Because of the special nature of the job, all night shift workers enrolled were male. The basic data indicated that night shift workers tended to be younger, shorter working years, but with higher BMI and longer hip circumference (*p* < 0.05). Night shift workers accompanied with a lower proportion of hypertension (HBP), diabetes (DM), and coronary artery disease (CAD) when compared with day workers (*p* < 0.05). We found night shift workers had a higher level of ALT, Cr, and UA as well (31.1 ± 17.1 mmol/L vs 30.2 ± 17.4 mmol/L, *p* = 0.019; 75.0 ± 13.2 mmol/L vs 73.8 ± 13.6 mmol/L, *p* < 0.001; 394.8 ± 81.1 mmol/L vs 391.0 ± 83.5 mmol/L,* p* = 0.032). The baseline characteristics of the participants were shown in Table 1.

**Table 1:** Basic characteristics of the study population

Characteristics	Night shift workers (n = 3008)	Day workers (n = 8015)	Overall	*p* value
Age (mean ± SD, y)	47.5 ± 3.5	49.4 ± 4.9	48.9 ± 4.7	< 0.001
Working years(mean ± SD, y)	28.6 ± 4.2	30.5 ± 5.7	30.0 ± 5.4	< 0.001
BMI (mean ± SD, kg/m^2^)	25.2 ± 3.1	24.8 ± 3.1	24.9 ± 3.1	< 0.001
Hip circumference (mean ± SD, cm)	96.1 ± 6.3	95.6 ± 6.1	95.7 ± 6.1	0.024
Smoking status (n, %)				0.599
Current smoker	1973 (65.6)	5250 (65.5)	7223 (65.5)	
Former smoker	263 (8.7)	659 (8.2)	922 (8.4)	
Never-smoker	772 (25.7)	2106 (26.3)	2878 (26.1)	
Alcohol consumption (n, %)				< 0.001
Current drinker	542 (18.0)	1980 (24.7)	2522 (22.9)	
Former drinker	46 (1.5)	191 (2.4)	237 (2.2)	
Never-drinker	2420 (80.5)	5844 (72.9)	8264 (75.0)	
HBP (n, %)	579 (19.2)	1728 (21.6)	2307 (20.9)	0.008
DM (n, %)	205 (6.8)	710 (8.9)	915 (8.3)	0.001
CAD (n, %)	12 (0.4)	98 (1.2)	110 (1.0)	< 0.001
SBP (mmHg)	126.7 ± 15.0	127.7 ± 16.5	127.4 ± 16.1	< 0.001
DBP (mmHg)	85.1 ± 11.9	83.8 ± 8.3	84.7 ± 11.7	< 0.001
TC (mmol/L)	5.1 ± 0.9	5.1 ± 0.9	5.1 ± 1.0	0.051
TG (mmol/L)	2.3 ± 2.1	2.3 ± 2.0	2.3 ± 2.1	0.693
LDL (mmol/L)	3.1 ± 0.7	3.1 ± 0.7	3.1 ± 0.7	0.478
HDL (mmol/L)	1.2 ± 0.3	1.3 ± 0.3	1.3 ± 0.3	< 0.001
hsCRP (mg/l)	2.4 ± 3.4	2.6 ± 4.3	2.5 ± 4.1	0.095
FBG (mmol/L)	5.9 ± 1.7	6.0 ± 1.0	6.0 ± 1.9	< 0.001
ALT (U/L)	31.1 ± 17.1	30.2 ± 17.4	30.5 ± 17.4	0.019
Cr (μmol/L)	75.0 ± 13.2	73.8 ± 13.6	74.1 ± 13.5	< 0.001
UA (mmol/L)	394.8 ± 81.1	391.0 ± 83.5	392.0 ± 82.9	0.032

BMI, body mass index; HBP, hypertension; DM, diabetes mellitus; CAD, coronary atherosclerotic heart disease; SBP, systolic blood pressure; DBP, diastolic blood pressure; TC, total cholesterol; TG, triglyceride; LDL, low density lipoprotein cholesterol; HDL, high-density lipoprotein cholesterol; hsCRP, hypersensitive C-reactive protein; FBG, fasting blood glucose

### Mean value and prevalence of each criterion for MetS

The study indicated that night shift workers had a lower level of SBP and FBG (126.7 ± 15.0 mmHg vs 127.7 ± 16.5 mmHg, *p* < 0.001; 5.9 ± 1.7 mmol/L vs 6.0 ± 1.0 mmol/L, *p* < 0.001). The level of DBP was higher in night shift workers than day workers (85.1 ± 11.9 mmHg vs 83.8 ± 8.3 mmHg, *p* < 0.001, Table 1). A total of 4759 participants had a diagnosis of MetS, the overall crude prevalence of MetS was 43.2%. 4979 participants had 1 or 2 components of MetS, while only 11.7% participants had none. Day workers seemed to have a higher proportion of abnormal blood pressure and blood sugar (61.9% vs 58.6%, 45.5% vs 40.8, *p* < 0.05). Night shift workers tended to have a higher proportion of low HDL (24.9% vs 22.6, *p* = 0.013). However, there was no significant difference in the prevalence of MetS between the two groups (42.6% vs 43.4%, *p* = 0.472, Table 2).

**Table 2:** Prevalence of each criterion for metabolic syndrome (n, %)

Characteristics	Nitht shift workers (n = 3008)	Day workers (n = 8015)	Overall (n = 11,023)	*p* value
Waist circumference (≥ 90 cm for men)	1304 (43.4)	3313 (41.3)	4617 (41.9)	0.056
Blood pressure (≥ 130/85 mm Hg or under medications)	1763 (58.6)	4963 (61.9)	6726 (61.0)	0.001
Fasting blood sugar (≥ 5.6 mmol/L or under medications)	1228 (40.8)	3646 (45.5)	4874 (44.2)	< 0.001
Triglycerides (≥ 1.7 mmol/L or under medications)	1612 (53.6)	4173 (52.1)	5785 (52.5)	0.153
High-density lipoprotein (< 1.04 mmol/L)	748 (24.9)	1813 (22.6)	2561 (23.2)	0.013
Metabolic syndrome (≥ 3 factors)	1282 (42.6)	3477 (43.4)	4759 (43.2)	0.472

### Age and working years stratified analysis of MetS

Due to the difference in age and length of service between night shift workers and day workers, we conducted a subgroup analysis by age and length of service stratification. The resulted indicated that the overall prevalence of MetS in 40–45, 45–50, 50–55 and ≥ 55 years old were 40.5%, 41.3%, 45.2%, 47.5%. The overall prevalence of MetS in 10–20, 20–25, 25–30, 30–35, and ≥ 35working years were 43.1%, 40.1%, 41.6%, 43.3% and 47.6%. However, no significant difference was found between night shift workers and day workers in all age groups and all working year subgroups (*p* ˃0.05, Additional file 1: Fig. S1).

### Associations between night shift workers and metabolic syndrome

We conducted an analysis of the association between NSW and MetS with its components. The univariate analysis revealed that there was no significant association between NSW and MetS (OR 0.97, 95% CI 0.89–1.06, *p* = 0.472, Table 3). This result was also confirmed after adjusting for age, working years, smoking status, alcohol consumption, and previous CAD (OR 1.03, 95% CI 0.94–1.12, *p* = 0.543). However, the adjusted model found that NSW was associated with SBP ≥ 130 mmHg (OR 1.11, 95% CI 1.02–1.21, *p* < 0.001) and Waist circumference ≥ 90 cm (OR 1.11, 95% CI 1.02–1.21, *p* < 0.001).

**Table 3:** Associations between NSW and METS with its components

Factors	Model 1		Model 2		Model 3		Model 4	
	OR (95% CI)	*p* value	OR (95% CI)	*p* value	OR (95% CI)	*p* value	OR (95% CI)	*p* value
SBP ≥ 130 mmHg	0.97 (0.89, 1.05)	0.446	1.08 (0.99, 1.18)	0.082	1.08 (0.99, 1.18)	0.082	1.11 (1.02, 1.21)	0.022
DBP ≥ 85 mmHg	0.88 (0.81, 0.96)	0.004	0.93 (0.85, 1.01)	0.078	0.93 (0.85, 1.01)	0.078	0.95 (0.87, 1.04)	0.262
SBP ≥ 130 mmHg and DBP ≥ 85 mmHg	0.98 (0.89, 1.07)	0.577	1.06 (0.97, 1.16)	0.192	1.06 (0.97, 1.16)	0.193	1.09 (1.00, 1.20)	0.05
Waist circumference ≥ 90 cm	1.09 (1.00, 1.18)	0.056	1.10 (1.01, 1.20)	0.037	1.10 (1.01, 1,19)	0.038	1.11 (1.02, 1.21)	0.017
TG ≥ 1.7 mmol/L	1.06 (0.98, 1.16)	0.153	1.02 (0.94, 1.11)	0.682	1.02 (0.93, 1.11)	0.694	1.02 (0.94, 1.11)	0.653
HDL-C < 1.04 mmol/L	1.13 (1.03, 1.25)	0.013	1.09 (1.00, 1.21)	0.084	1.09 (0.99, 1.21)	0.084	1.06 (0.96, 1.18)	0.227
DM or FBG > 5.6 mmol/L	0.83 (0.76, 0.90)	< 0.001	0.94 (0.86, 1.02)	0.136	0.94 (0.86, 1.02)	0.135	0.96 (0.88, 1.04)	0.304
METS	0.97 (0.89, 1.06)	0.472	1.01 (0.93, 1.10)	0.826	1.01 (0.93, 1.10)	0.833	1.03 (0.94, 1.12)	0.543

Model 1 No adjusted

Model 2 Adjusted for age

Model 3 Adjusted for age and working years

Model 4 Adjusted for age, working years, smoking status, alcohol consumption, and previous CAD

SBP, systolic blood pressure; DBP, diastolic blood pressure; BMI, body mass index; TG, triglyceride; HDL-C, high-density lipoprotein cholesterol; DM, diabetes mellitus; FBG, fasting blood glucose; METS, Metabolic syndrome

## Discussion

This cross-sectional study examined the association between long-term NSW and metabolic syndrome and found that long-term NSW was not associated with an significantly increased risk of MetS. Night shift workers tended to have a higher proportion of low HDL when compared with day workers. No significant difference was found between night shift workers and day workers in all age groups and all working year subgroups.

Pervious study concentrated on nurses indicated that a longer duration of rotating NSW was associated with an increased risk in CHD [15], our study focuses on another special character, train drivers, who work the same round-the-clock shifts. Our present study showed the overall crude prevalence of metabolic syndrome was 43.2%, which was higher than previous studies [7, 22]. This result may be related to the high incidence of hypertension and diabetes in China, which is currently in the third to the fourth stage of epidemiology [23, 24]. Our study suggested that shift workers had a higher level of BMI and hip circumference, which was consistent with previous studies [25, 26]. However, night shift workers had a lower proportion of history of chorionic diseases such as hypertension, DM, and coronary heart diseases. So the influence of NSW on MetS could be quite important.

The association between NSW and MetS can be explained by the following reasons. First, it is reported that NSW leads to sleep loss and increases the risk of obesity and diabetes [27]. On the other hand, irregular food intake influence energy balance and weight regulation, which lead to metabolic disturbance [28, 29]. Second, circadian disruption and eating meals irregularly disturb the natural rhythmicity of insulin action and lead to insulin resistance and obesity [30–32]. Third, it is reported that the gene REV-ERBα, which regulates circadian rhythms, is associated with liver lipid metabolism, which influence metabolic disturbance [33, 34]. Therefore, NSW are key regulators of metabolic disturbance, which can affect metabolic syndrome.

NSW may result in a higher risk of liver disfunction and non-alcoholic fatty liver disease [20, 35], our present study consisted of the previous results. Shift work also has a confluence on renal function according to our study, shift workers showed a higher level of creatinine and uric acid. Animal study suggest that circadian rhythm reversals may affect renal metabolic rhythms, which may result in impaired kidney function [36]. However, no significant differences in TC, TG, and LDL-C were found between groups. Previous studies found that NSW may lead to higher risk of dyslipidemia and related to a higher risk of cardiovascular diseases [37, 38]. The association between shift work and lipid may need further studies.

Our study indicated that railway workers had a high prevalence of waist circumference increase, hyperglycemia, and high triglycerides over 40%, while the prevalence of hypertension was 60%. The railway workers are in poor health. Night shift workers had a higher prevalence of waist circumference increase and low LDL-C when compared with day workers, which was consistent with the previous study [15]. Hypertension and hyperglycemia were more common among day workers, which may be related to different lifestyles. However, the overall prevalence of metabolic syndrome of night shift workers and day workers showed no significant difference.

As for the difference of age and working years between shift workers and day workers, we did a subgroup analysis of the study. The finding suggested that no significant difference was found between night shift workers and day workers in all age groups and all working year subgroups. The prevalence of metabolic syndrome was increased by age and working years, which was consistent with previous studies [6, 39]. We did the analysis between night shift workers and metabolic syndrome and its components, the result insisted that NSW was not associated with a higher risk of metabolic syndrome. We reviewed the studies and found that the previous results were inconsistent. Some studies have suggested that shift work and sleep quality may be associated with an increased risk of metabolic syndrome [6, 12, 15], while others were not [14, 40]. The results of the study by Vetter C et al. suggested that recent night shift work may be more related to the onset of cardiovascular diseases [15]. It is not clear whether the results were affected by years of work, our study indicated that long-term shift work was not associated with the increased risk of metabolic syndrome. Further study may concentrate on recent shift work. After adjusting for age, working years, smoking status, alcohol consumption, and history of chronic diseases, our analysis showed that NSW was associated with elevated systolic blood pressure, and waist circumference increase. The treatment of metabolic syndrome was based on the diseases, studies showed exercise training, mediterranean diets, and supervised lifestyle intervention may improve outcomes and reduce individual risk factors of metabolic syndrome [41–43]. Our study insisted that railway workers need long-term and effective interventions to reduce the incidence of metabolic syndrome.

## Strengths and limitations

This study investigates the relationship between long-term night shift workers and MetS and has some limitations. First, since there are more men working along railway lines, our study lacks data on women. Secondly, the data on physical activity, sleep quality and duration, nutritional status, and exposure to noise were absent, which may have effect on the results of analysis. Third, because we are an observational study and based on physical examination data, we lack data on the treatment of the disease. However, despite the above limitations, we still believe that this study can reflect the association between long-term night shift workers and metabolic syndrome. Although there is no significant difference between shift work and the incidence of metabolic syndrome, the prevention and treatment of metabolic syndrome and its factors still need to be carried out in all railway workers.

## Conclusion

Long-term night shift workers had a higher prevalence of MetS. However, long-term NSW is not associated with a significantly increased risk of metabolic syndrome in male railway workers in southwest China. Long-term NSW is associated with elevated SBP, and waist circumference increase. All the railway workers need long-term and effective interventions to reduce the incidence of MetS.

## Supplementary Information

**Additional file 1:** **Fig. S1.** Age and working years stratified analysis of MetS.

## Data Availability

The data that support the findings of this study are available from the Affiliated Hospital of Chengdu Medical University, but restrictions apply to the availability of these data, which were used under license for the current study, and so are not publicly available. Data are however available from the authors upon reasonable request and with permission of Affiliated Hospital of Chengdu Medical University.
