# Supplementary figures and images for: The association between long-term night shift work and metabolic syndrome: a cross-sectional study of male railway workers in southwest China
Source: BMC Cardiovasc Disord. 2022 Jun 11;22:263. doi: 10.1186/s12872-022-02705-7 (PMC9188692; doi:10.1186/s12872-022-02705-7)

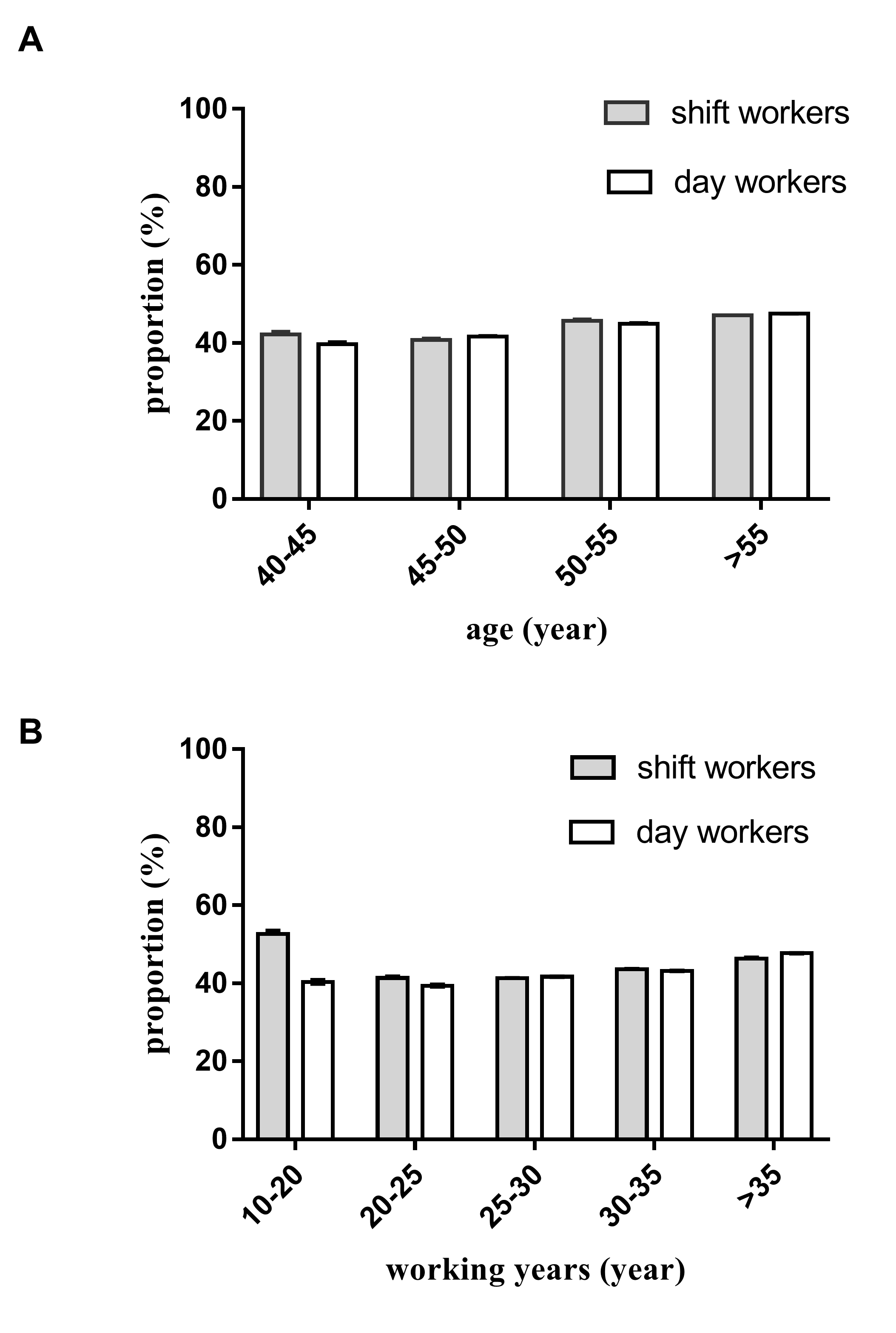

Supplement: Supplementary file 1 — Additional file 1: Fig. S1. Age and working years stratified analysis of MetS. [file 12872_2022_2705_MOESM1_ESM.tif]
